# Burkholderia Cepacia Causes Frontal Mucopyocele with Anterior Cranial Fossa Extension: A Novel Case Report

**DOI:** 10.22038/ijorl.2021.51654.2753

**Published:** 2021-09

**Authors:** Athanasios Saratziotis, Claudia Zanotti, Maria Baldovin, Vlatko Prosenikliev, Enzo Emanuelli

**Affiliations:** 1 *Department of Otolaryngology, General University Hospital of Larisa, Greece* *.*; 2 *Department of Neurosciences, Otolaryngology Section, University of Padua, Italy* *.*; 3 *Department of Otolaryngology, General Hospital San Dona di Piave, Venice, Italy* *.*

**Keywords:** Burkholderia cepacia complex, Mucocele, Frontal Sinus, Surgical Endoscopy, Culture Media

## Abstract

**Introduction:**

Burkholderia cepacia complex (Bcc) is a group of gram-negative bacilli that have rarely been isolated in the ear, nose and throat region in immunocompetent patients. Bcc show resistance to most available antibacterial drugs.

**Case Report:**

We present the case of an immunocompetent 31-year-old male reporting a pulsating headache with right supraorbital swelling associated with exophthalmos. A brain CT scan showed an expansive giant cystic lesion occupying the right frontal sinus, extending to the anterior cranial fossa. Management and outcome: drainage with the resecting of the floor of the frontal sinus from the orbital plate of the ethmoid bone to the nasal septum (Draf IIb) was performed with wide marsupialization of the mucopyocele. Polymerase chain reaction-restriction fragment length polymorphism (PCR-RFLP) analysis was used to identify the isolate. MRI 1 and 12 months after surgery showed complete lesion removal. The patient was followed for 12 months with complete recovery of symptoms.

**Conclusion:**

Paranasal sinuses disease with cranial expansion and orbital complications constitutes an emergency. For the first time in the literature*, *Bcc was isolated in the frontal sinus, extending into the anterior cranial fossa, in an immunocompetent patient. An endoscopic surgical approach with microbiological identification and management by appropriate antibacterial drug treatment seems to be the key to success.

## Introduction

Burkholderia cepacia complex (Bcc) is a group of phenotypically similar but genetically distinct, motile, glucose-non-fermentative, gram-negative bacilli that can be found in soil or water. Bcc bacteria have a highly versatile metabolism, a multireplicon genome structure, are capable of adapting at a rapid rate by mutation, and are widely distributed in the environment. One of the main problems associated with Bcc bacteria is their intrinsic resistance to many common antibiotics and antiseptics, and their capacity to acquire resistance against many more. B. cepacia has repeatedly been isolated in patients with cystic fibrosis (1) and chronic granulomatous disease, and in immunosuppressed patients, often in association with other germs (2). In a few reports, Bcc has already been isolated in nasal mucosa of immunocompetent patients with chronic rhinosinusitis (3). There is evidence that hospital outbreaks have been caused by contamination of medical equipment and disinfectants, while in hospital patients without cystic fibrosis, Bcc causes infection through the bloodstream. With a mortality rate estimated at 25–64%, it is now considered an emerging pathogen. It is highly resistant to many antibiotics, and therefore is often difficult to treat (4). 

B. cepacia complex comprises nine officially recognized species groups. However, their phenotypic identification is currently challenging. By way of a solution to this problem, 16S rRNA and recA gene sequence analysis has improved identification (5). Bcc species exhibit similar properties to other opportunists, such as Pseudomonas species. Τhey have the capacity to survive in conditions where nutrients are scarce, metabolising, for example, the organic matter present in aquatic environments, where survival and proliferation are possible and can even use certain antibacterial drugs as sources of carbon (6). Paranasal sinus mucoceles (PSM) are benign cystic lesions surrounded by respiratory epithelium and containing sterile mucous, resulting from obstruction of the natural ostium of the paranasal sinuses (7). 

The condition most commonly affects the frontal sinus and the ethmoidal cells and is more frequent in adult patients between the ages of 40 and 60, regardless of gender (8,9). A mucocele can remain asymptomatic for a long time. Typical presenting symptoms are headache, sense of pressure or swelling of the face, respiratory nasal obstruction, and rhinorrhoea (10). 

If there is orbit involvement, the manifestation generally involves pain, proptosis, diplopia, dislocation of the bulb, and blurring or reduction of visual acuity. Intracranial extension may cause meningitis or predispose to the formation of a cerebrospinal-fluid (CSF) leak. The risk of complications, especially orbital ones, is greater when there is an overlapping infection, resulting in faster growth of the mucocele, in this case called a mucopyocele (11,12). A suspected diagnosis of mucopyocele on nasal endoscopy can be confirmed by computed tomography (CT) scan and magnetic resonance imaging (MRI).

## Case Report

In July 2017, a 31-year-old male presented to our department with palpebral and right supraorbital swelling associated with nasal obstruction, posterior rhinorrhoea, pulsating headache and exophthalmos. The patient, from Venice, Italy, had worked as a high-school teacher since the age of twenty-five. The symptoms had first appeared one month earlier, and the right exophthalmos had progressively worsened, despite antibiotic therapy with amoxicillin/clavulanic acid and oral corticosteroid. 

In 2008, the patient had undergone endoscopic sinus surgery and septoplasty at a different centre for chronic rhinosinusitis with nasal polyps. The patient was immunocompetent, and not suffering from cystic fibrosis or other chronic diseases.

**Investigations**

On admission, the endoscopic evaluation revealed scar tissue between the middle turbinate and the lateral wall of the right nasal fossa, with purulent drain from the frontal recess; no nasal polyps were found.

**Fig 1:**
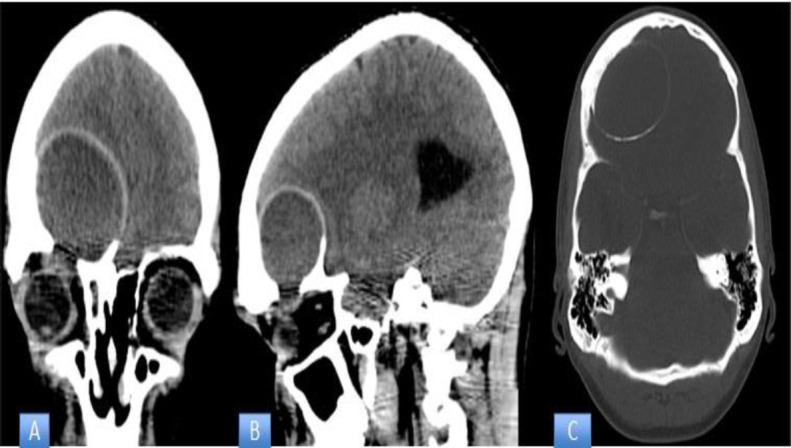
CT scan in a coronal, B sagittal and C axial planes. Mucopyocele of the roof of the right orbit, with calcified walls and extension to the anterior cranial fossa displacing the adjacent brain tissue. The area of the mucopyocele is indicated with a black arrow

An emergent brain CT scan showed an expansive cystic lesion of about 50 x 40 mm with calcified walls without contrast enhancement, occupying the right frontal sinus, with partial erosion of the orbital roof and extending to the anterior cranial fossa (shown in Figure 1).

**Treatment**

**Fig 2:**
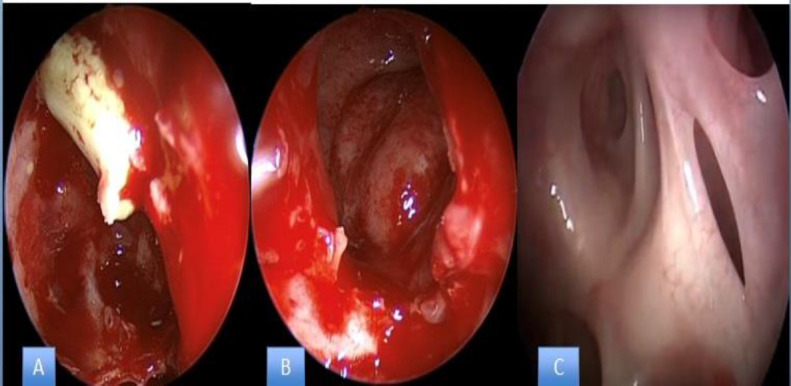
Endoscopic view with 45˚ endoscope in the frontal recess during endoscopic procedure Draf 2b. A. Appearance of mucopyocele B. After marsupialization in the frontal sinus C. One year after, follow up view. The area of the mucopyocele is indicated with a black arrow

Emergent endoscopic sinus surgery (ESS) was scheduled in order to treat the expansive frontal sinus mucocele. Under general anaesthesia, the patient was placed supine in an anti-Trendelenburg position and both nostrils were decongested with cottonoid soaked in a solution of adrenaline 1:100,000 with 2% lidocaine. A frontal sinusotomy was then performed, drilling out the frontal sinus floor between the lamina papyracea laterally and the nasal septum medially (Draf IIb), shown in Figure 2A). After incision of the cystic lesion capsule, the purulent content was drained and the mucocele was widely marsupialized in order to prevent reaccumulation (shown in Figure 2B). Nasal packing with absorbable oxidized regenerated cellulose was placed into the frontal sinus. 

Purulent material was plated onto sheep blood agar (COS; bioMérieux, Marcy l’Etoile, France) and chocolate agar with PolyVitex (bioMérieux). Gram staining was performed. The isolates were identified as Bcc using a commercial test (Vitek GN, bioMérieux), and the identification was confirmed by the ID32 GNI test (bioMérieux).Additionally, *polymerase chain reaction-*restriction *fragment length polymorphism* *(PCR-RFLP)* analysis was used to identify the isolate. Antibiotic susceptibility testing was performed using a microdilution method (Sensititre panel; Biomedical, Scorzè, Venice, Italy) and confirmed by the E-test (AB Biodisk, Solna, Sweden). Microbiological examination of the specimen showed Burkholderia contaminans growth, resistant to amoxicillin/clavulanic acid, ampicillin sulbactam and ciprofloxacin, but sensitive to levofloxacin, administered postoperatively for 8 days. Histopathological examination of the tissue obtained from the left frontal sinus revealed: pseudostratified columnar epithelium predominated, associated with areas of squamous and cuboidal epithelium. The cellular infiltrate of neutrophils, lymphocytes and plasma cells was consistent with chronic inflammation. The surrounding bone showed osteoblastic activity with osteoid and sclerosis alternating with areas of active bone destruction.

**Fig 3:**
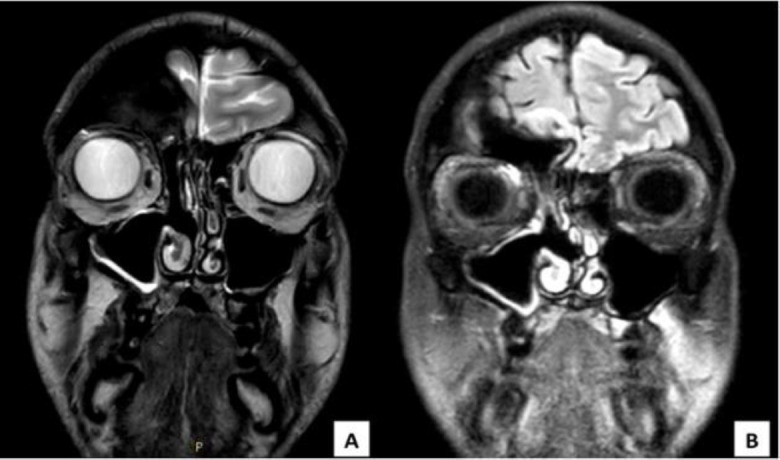
Coronal view of 10 months MRI follow-up weighted in T2 (A) and T1 with gadolinium (B). The right frontal and maxillary sinuses appear completely aerated. The previous area of the mucopyocele is indicated with a black arrow

Two days after the procedure, the patient’s exophthalmos and headache already showed satisfactory improvement, and he was discharged six days later with no visual, neurological or systemic complication. Contrast-enhanced MRI performed one month after surgery showed complete lesion removal without evidence of recurrence (shown in Figure 3). At the same point in time, i.e. one month after the procedure, the exophthalmos was completely resolved. The last endoscopic evaluation, performed 12 months after surgery, was still negative, as was a control MRI performed after 12 months (shown in Figure 2 C). 

## Discussion

There are sporadic reports of* B. *cepacia infections in immunocompetent patients. However, in several cases of pseudo-epidemics and nosocomial infections, contaminated disinfectant and anaesthetic solutions have been cited as causes. Moreover, several hospital outbreaks have been caused by these bacteria over the last twenty years, owing to their contamination of sterile pharmaceuticals such as intravenous drugs and solutions, and non-sterile nasal sprays, mouthwash, pre-operative skin solutions and hand sanitizers, and water-based products in general). Antibacterial drugs and disinfectants, and especially biocides used in the formulation of pharmaceuticals, do not impact on the spread of Bcc bacteria and recurrent contamination episodes (5).

The pathogenesis of mucopyoceles of the paranasal sinuses is still not entirely known. Two main etiopathogenetic mechanisms are likely to be involved: (i) the obstruction of the natural ostium of the paranasal sinuses, and (ii) chronic inflammation associated with conditions such as chronic, allergic or non-allergic rhinosinusitis and mucociliary clearance defects (i.e., cystic fibrosis) (13). Nasal polyposis has been proven to be a predisposing factor (8). Other diseases that have been described are previous nasopharyngeal cancer treated by radiotherapy, chronic nasal infection due to Staphylococcus spp. and Klebsiella pneumoniae subsp. rhinoscleromatis (14). According to the literature, no significant role is played in the etiopathology of sinonasal polyposis by fungal agents (15). However, one third of cases remain idiopathic even in the adult population. Some authors suggest that Aspergillus fumigatus is the most frequent causative agent of chronic rhinosinusitis and others that Aspergillus flavus is the most common agent of fungal rhinosinusitis after a study of a population in Iran (16). The isolation of Neoscytalidium dimidiatum from a case of eosinophilic fungal rhinosinusitis (EFRS) has also been described (17).Mucoceles are slow-growing formations, but their expansion in the sinus cavities can cause remodelling and erosion of the bony walls, with dislocation of the surrounding structures, particularly the orbit content and even intracranial invasion if the thin posterior wall of the frontal sinus is eroded, causing potentially dangerous complications such as epidural abscess, subdural empyema, CSF leak, and meningitis (10,13,18). 

The term “giant” is commonly used in cases with intracranial and/or intraorbital extension, with prominent mass effect. Given that the content of the lesion was purulent, we can state that our patient was affected by a giant frontal mucopyocele. 

The management of mucopyoceles has been widely described and it is now universally recognized that surgical treatment involves the marsupialization of the lesion, which ensures minimal morbidity while at the same time preventing recurrence. The technique consists in opening and draining the mucocele, keeping the cavity in wide communication with the nasal fossa. Generally, it would be preferable not to operate under acutely infectious conditions; however, an exception is made for acute symptomatic mucoceles, especially if complicated, e.g. by exophthalmos, or when unresponsive to antibacterial treatment, when surgery is urgently required. Moreover, the appropriate material will be available to identify the species of Bcc for targeted pharmaceutical treatment. 

Given the alarming appearance of the symptoms, chiefly the exophthalmos but also the evidence of the CT scan showing an expansive cystic lesion of about 50 x 40 mm, the decision was made to proceed directly to surgery. Moreover, the patient had already started antibiotic treatment and his clinical picture was deteriorating, while the exact identification of the pathogen gave us the possibility of targeted antibiotic therapy.

For targeted antibiotic therapy, it is necessary to isolate the bacteria. The intrinsic resistance of Bcc to aminoglycosides, 1^st^ and 2^nd^ generation cephalosporins, anti-pseudomonal penicillin, amoxicillin/clavulanic acid, ampicillin, ampicillin/sulbactam, cephalothin, and cefoxitin has previously been confirmed. When B. cepacia complex grows in blood cultures, ceftazidime, piperacillin-tazobactam and meropenem are the active antibacterial agents of choice (19). 

Burkholderia cepacia complex infections have rarely been reported in immunocompetent patients, but have been isolated in patients with chronic rhinosinusitis, without cystic fibrosis, chronic granulomatous disease or other immunosuppression conditions (20). 

In our case, we isolated the Burkholderia contaminans bacterium, belonging to the Burkholderia cepacian complex (Bcc), which is a group of gram-negative bacteria that can cause serious respiratory infections in immunosuppressed individuals. In particular, Bcc infection in patients with cystic fibrosis is associated with a worse prognosis and early death (21). 

**Risk factors**

Our patient was not subject to risk factors contributing to the growth of the bacterium. However, the following considerations must be taken into account: he had previously undergone endoscopic surgery and for many years had performed intranasal washing and used nasal sprays. As in the case of mucocele, the probability of colonization by Bcc bacteria and other pathogens was higher because mucous secretions accumulate as a result of a restrictive sinus environment (6). 

One study investigates the fear that Bcc in water-based pharmaceutical products poses a risk of contamination. Additionally, they present an inherent resistance to antibiotics and antiseptics (5).

Twenty-two per cent of non-sterile product recalls between 1998 and 2006 were due to B. cepacia, according to data from the U.S. Food and Drug Administration (FDA). In the ensuing years this trend increased, so that for 2004 to 2011, the corresponding figure was 34% (6). Under normal circumstances, mucociliary activity in the respiratory tract clears it of Bcc strains, so respiratory infection by Bcc is a rare occurrence in healthy individuals with a normal immune response. However, in the case of nosocomial infection of patients without cystic fibrosis, venous and urinary catheters, mechanical ventilation with the use of endotracheal tubes, hemodialysis, and long periods spent in intensive care are among the most commonly identified risk factors (3-6,19).

A 2014 meta-analysis on surgical management of frontal and fronto-ethmoid mucoceles included 31 studies divided into historical (425 mucoceles) and contemporary (542 mucoceles) groups. It revealed that a significantly greater percentage of endoscopic approaches were utilized in the contemporary cohort than in the historical one (53.9% vs. 24.7%). Additionally, the contemporary cohort had a significantly smaller percentage of external approaches than the historical one. The percentage of combined cases taken into account was similar. These results show that a large proportion of frontal and fronto-ethmoid mucoceles are now being managed endoscopically. Major complication rates and overall recurrence were similar between the cohorts, confirming that the wider adoption of endoscopic techniques does not compromise surgical efficacy (22).

Despite the increased use of endoscopic surgery in the last twenty years, open procedures are still used depending on the size and extension of the lesion. A recent systematic review (23) considering 85 cases of giant frontal mucoceles with significant intracranial extension reported that 65.9% of patients were managed with an open approach and only 34.1% with a strictly endoscopic technique. However, it is important to note that the majority of endoscopic cases (90%) date from after 2004, as opposed to only 40% of external cases. Furthermore, despite the fact that only one-third of these patients were treated endoscopically, the review found that an endoscopic procedure with extensive marsupialization was safe and effective even for this kind of mucocele. In terms of surgical complications, no significant difference was found between endoscopic and open approaches (22), though Stokken et al. have reported that major complications (intracranial abscess, meningitis, CSF leak) were at a significantly higher level for the latter (23).

Post-operative targeted intravenous antibiotic therapy is crucial and involves the use of nasal irrigation and topical steroids. Close endoscopic follow-up should be adjusted, until full recovery is achieved.

## Conclusion

Paranasal sinuses disease with cranial expansion and orbital complications constitutes an emergent situation. For the first time in the literature, we present a novel case with a giant mucopyocele in the frontal sinus extending to the anterior cranial fossa caused by Bcc in an immunocompetent patient. An endoscopic surgical approach with wide marsupialization combined with microbiological identification and management by appropriate antibacterial treatment seems to be the key to success. In-vitro antibiotic susceptibility data, established clinical responses and personal expertise will provide physicians with the necessary tools to improve each patient’s individual assessment. 
